# Supplementary material for: Locat: Joint enrichment and depletion testing identifies localized marker genes in single-cell transcriptomics
Source: bioRxiv. 2026 Apr 9:2026.04.03.716370. Preprint. [Version 2] doi: 10.64898/2026.04.03.716370 (PMC13081944; doi:10.64898/2026.04.03.716370)
Supplement: Supplement 1 [file media-1.pdf]

Supplementary Information

Contents

S1 Supplementary Methods **2**
S1.1 Weighted Gaussian Mixture Models (WGMMs) 2
S1.1.1 Model. 2
S1.1.2 Initialization. 3
S1.1.3 Component selection via BIC. 3
S1.1.4 Heuristic selection of per-gene component count 3
S1.2 Empirical null distribution calibration as a function of sample size. 4
S1.3 Global correction and empirical calibration 4
S1.4 Stability of the Adjusted Localization Score 4
S1.4.1 Sensitivity penalty (α_{sens}). 5
S1.4.2 Prevalence penalty (α_{size}). 5
S1.5 Operational analysis defaults 5
S1.6 Robustness to Embedding Choice and Random Seed 5
S1.7 Computational complexity and runtime profiling 6
S1.8 SpectralRH 7
S1.8.1 Design gap in existing methods 7
S1.8.2 Algorithm 8

S2 Supplementary Data **9**

S3 Supplementary Figures **10**

S4 Supplementary Tables **24**

S5 Supplementary Notes **28**

S1 Supplementary Methods

S1.1 Weighted Gaussian Mixture Models (WGMMs)

A Gaussian mixture model (GMM) assumes observations are generated from a finite mixture of Gaussian distributions with unknown parameters. A weighted GMM (WGMM) extends this model by incorporating sample weights that encode the relative importance of observations.

S1.1.1 Model.

The probability density function of a K-component WGMM is a weighted sum of K Gaussian component densities:

$$f(\mathbf{x}) = \sum_{k=1}^{K} \pi_k \, \mathcal{N}(\mathbf{x} \mid \mu_k, \Sigma_k), \tag{1}$$

where $\mathbf{x}$ is a point in sample space and K is the number of components. Here, $\pi_k \geq 0$ is the mixture weight of component k with $\sum_{k=1}^{K} \pi_k = 1$, and μ_k and Σ_k are that component's mean and covariance, respectively. In the main Methods, the background density $f_0(\mathbf{x})$ and gene-specific density $f_g(\mathbf{x})$ are both instantiated as WGMMs under this formulation.

Model estimation via weighted EM updates. Model fitting requires estimation of (π_k, μ_k, Σ_k), performed iteratively using the Expectation-Maximization (EM) algorithm.

In the notation below, we omit iteration indices; the symbol $\leftarrow$ denotes parameter updates.

Given parameter estimates at iteration t, (π_k, μ_k, Σ_k), the E step computes responsibilities:

$$r_{ik} = \frac{\pi_k \, \mathcal{N}(\mathbf{x}_i \mid \mu_k, \Sigma_k)}{\sum\limits_{\ell=1}^{K} \pi_\ell \, \mathcal{N}(\mathbf{x}_i \mid \mu_\ell, \Sigma_\ell)}, \tag{2}$$

which are the posterior probabilities that cell i belongs to component k.

Sample weights s_i enter the M step through effective weighted responsibilities:

$$v_{ik} = s_i \, r_{ik}, \tag{3}$$

such that higher weighted cells exert proportionally stronger influence.

Let $w_k = \sum_i v_{ik}$ and $u = \sum_{k=1}^{K} w_k$. The mixture parameters are then updated as

$$\pi_k \leftarrow \frac{w_k}{u}, \tag{4}$$

$$\mu_k \leftarrow \frac{\sum_i v_{ik} \mathbf{x}_i}{w_k}, \tag{5}$$

$$\Sigma_k \leftarrow \frac{\sum_i v_{ik}(\mathbf{x}_i - \mu_k)(\mathbf{x}_i - \mu_k)^\top}{w_k} + \lambda I, \tag{6}$$

where λ is a small regularization term to ensure numerical stability. These updates match the standard weighted-EM formulation, and our implementation follows Gebru et al. (2016) [1].

S1.1.2 Initialization.

In our implementation, component means are initialized with weighted k-means++ (using $\{s_i\}$ as sample weights), and covariances are initialized as scaled identity matrices. Covariance regularization is applied through `reg_covar` in `locat_condensed.py`, with default scale

$$\lambda = \max\left(\frac{\text{min\_dist}^{2/d}}{6}, 10^{-4}\right),$$

where min_dist is the mean nearest-neighbor distance in the embedding and d is embedding dimensionality.

Multiple initializations are run in parallel, and the solution with the largest weighted log-likelihood is retained.

S1.1.3 Component selection via BIC.

To determine mixture complexity (hyperparameter K), we use the Bayesian Information Criterion (BIC), which balances fit quality against model complexity through a parameter-count penalty. In `locat_condensed.py`, BIC search is run at two waypoints: full sample size and $\sqrt{n}$ sample size. Candidate component counts start at $K = 1$, and the search range is expanded when the minimum occurs near the current upper boundary. The selected waypoint values are then interpolated to estimate component count as a smooth function of sample size.

S1.1.4 Heuristic selection of per-gene component count

In the LOCAT workflow, a `LOCAT` object is instantiated with expression data and embedding coordinates. For each dataset, a background WGMM over all cells is fit once and reused across gene-wise scanning (e.g., `gmm_scan_new`). This model partitions the embedding into K_0 mixture components, each representing a manifold region. For each gene, we then select a gene-specific component count K_g based on how that gene's weighted cells are distributed across the background components.

Specifically, for each background component we compute the fraction of the gene's total weight assigned to that component (via background responsibilities). We sort these fractions in decreasing order and choose the smallest number of components whose cumulative fraction exceeds a fixed threshold (e.g., 95%). That number defines K_g for the gene's WGMM. Genes concentrated in a few compact regions receive small K_g, whereas genes spread across multiple regions receive larger K_g.

S1.2 Empirical null distribution calibration as a function of sample size.

Given a raw concentration statistic z, we calibrate it with a Monte Carlo null that is matched to gene sample size.

We define a grid over the fraction of non-zero entries in the sample weight vector:

$$p \in \{p_1, \ldots, p_L\} \subset \big[10/n,\, 1\big], \tag{7}$$

where n is the total number of data points and L is the number of grid points.

Then, for each value of p, we repeat the following steps many times:

1. Randomly generate a binary weight vector $\hat{s} \in \{0, 1\}^n$ with np non-zero entries.
2. Fit a WGMM using exactly the same pipeline used for real genes.
3. Compute the same raw statistic z.

This gives a null distribution of z at each p. From that distribution, we estimate the null mean $\mu(p_\ell)$ and null standard deviation $\sigma(p_\ell)$. To evaluate these quantities at arbitrary sample sizes, we fit piecewise cubic Hermite interpolating polynomials (PCHIP) to $\{\mu(p_\ell)\}$ and $\{\sigma(p_\ell)\}$, yielding continuous functions $\mu(p)$ and $\sigma(p)$.

For a real gene with observed proportion p of non-zero weights and raw statistic z, we report the calibrated score

$$Z_{\text{null}}(p; z) \;=\; \frac{z - \mu(p)}{\sigma(p)}, \tag{8}$$

which expresses z in standard-deviation units relative to the null at that sample size.

S1.3 Global correction and empirical calibration

In Locat, per-gene localization tests are not an exchangeable family of independent tests. Genes differ in prevalence, detection noise, and effective sample size, and each final score combines concentration and depletion components with distinct null behavior. Gene-level statistics are also coupled through shared embedding and model fitting, so the pipeline is not equivalent to i.i.d. draws from one null family. Therefore, we do not use a single genome-wide correction as the primary calibration strategy. Instead, we assess calibration empirically using simulation-based null analyses matched to the pipeline structure and prevalence regime (Supplementary Fig. S1).

S1.4 Stability of the Adjusted Localization Score

The adjusted localization score (Eq. 20) includes two penalty coefficients, α_{size} and α_{sens}, modulating the prevalence and sensitivity components, respectively. Across all datasets we used fixed values $\alpha_{\text{size}} = 0.05$ and $\alpha_{\text{sens}} = 0.12$, selected once based on preliminary simulations and not tuned per dataset.

S1.4.1 Sensitivity penalty (α_{sens}).

Fig. S17(A–B) illustrates the effect of α_{sens} using simulated genes with increasing bleed fraction (fraction of expressing cells outside the primary enriched region). Panel S17A shows representative simulated patterns across the bleed range, and Panel S17B summarizes the resulting $-\log_{10}$ adjusted p-values across simulations.

In structured embeddings, many genes show some autocorrelation relative to a weak or random null, so the concentration test can assign similarly small p-values to many genes. α_{sens} refines ranking in this regime by downweighting genes with expression outside enriched regions, thereby reordering genes by bleed. Rankings remain highly stable across $\alpha_{\text{sens}} \in [0.05, 0.30]$ (Spearman $\rho > 0.9998$).

S1.4.2 Prevalence penalty (α_{size}).

Supplementary Fig. S17(C–D) shows the effect of α_{size} across simulated genes spanning a range of prevalences and signal strengths. Panel S17C shows representative simulated patterns across the prevalence range, and Panel S17D summarizes the resulting $-\log_{10}$ adjusted p-values.

Increasing α_{size} lowers adjusted scores across genes, with a larger reduction for low-prevalence genes than for higher-prevalence genes (Panel S17D). This term primarily separates genes with comparable enrichment strength but differing numbers of expressing cells, and prevents very rare genes from filtering to the top when many genes exhibit similarly strong concentration signal. Rankings remain stable across $\alpha_{\text{size}} \in [0.1, 0.2]$ (Spearman $\rho > 0.996$).

S1.5 Operational analysis defaults

To ensure reproducibility and avoid ambiguity between implementation-level function defaults and manuscript analysis settings, Table S1 lists the exact parameter values used in the primary Locat analysis path (`gmm_scan_new`) unless otherwise stated.

Unless explicitly overridden, background density estimation uses uniform per-cell weights in the primary analysis.

S1.6 Robustness to Embedding Choice and Random Seed

To assess robustness of Locat 0.1 in the IFN-β-stimulated PBMC dataset, we split stimulated cells into two disjoint one-third subsets and reran preprocessing independently within each subset, including PCA recomputation. We then ran Locat on embeddings defined by 4 PCs, 8 PCs, 12 PCs, and a BiPCA-based setting in which the effective rank was estimated separately within each fold, that number of PCs was used to construct the neighbor graph, and a UMAP embedding was then computed for Locat. For each fixed embedding, Locat was rerun with three random seeds so that seed variability could be separated

from split-to-split variability. Tables S3–S5 summarize cross-fold reproducibility, within-fold seed stability, and embedding sensitivity for these analyses.

S1.7 Computational complexity and runtime profiling

For the current implementation in `locat_condensed.py`, runtime is naturally split into two phases:

$$T_{\text{total}}(n, G) \; = \; T_{\text{setup}}(n) + T_{\text{scan}}(n, G). \tag{9}$$

Setup phase. `T_setup` includes background-model preparation and concentration-null calibration. In the current code path, setup may access full pairwise cell distances during regularization and component-support heuristics. As a result, setup scaling with cell count is superlinear and can include a quadratic term in n. For fixed embedding dimension and fixed runtime hyperparameters, setup is therefore primarily cell-count dominated.

Scan phase. `T_scan` runs `gmm_scan_new` across genes. For fixed embedding dimension and bounded mixture complexity, scan runtime grows approximately linearly with the number of tested genes G. It also increases with n because per-gene density evaluation and model fitting become more expensive.

Runtime profiling protocol. To make implementation-level scaling explicit and reproducible, we benchmarked runtime on real data from the IFN-β-stimulated PBMC dataset by subsampling cells and genes over predefined grids.

1. Load one real dataset and fixed embedding coordinates.
2. For each (n, G) pair, subsample n cells and G genes without replacement.
3. Measure setup time separately (background fit + null calibration).
4. Measure scan time separately (`gmm_scan_new` over selected genes).
5. Repeat each (n, G) condition across multiple random seeds and report median wall-clock time.

Benchmarking was run on a system using an AMD EPYC 7282 16-Core CPU and NVIDIA RTX A6000 GPU.

Empirical scaling is shown in Fig. S18. Panel A (runtime versus number of genes at fixed n) shows near-linear growth of scan time with G, while setup time is comparatively stable across G at fixed n. Panel B (runtime versus number of cells at fixed G) shows that setup increases steeply with n, and scan also increases with n across all gene-grid settings.

S1.8 SpectralRH

S1.8.1 Design gap in existing methods

SpectralRH was designed to address a specific methodological gap in existing gene localization methods. We believe that our analyses of the advantages of our dual concentration-depletion criterion would not be complete without addressing this gap.

Existing gene localization methods detect spatial patterns through distinct but complementary approaches.

- Hotspot quantifies local spatial autocorrelation via Geary's C, identifying genes with correlated expression among neighboring cells.
- LMD measures diffusion rates on the cell graph, scoring genes whose expression is concentrated in compact regions where random walks remain localized.
- Haystack compares kernel density estimates in low-dimensional spatial coordinates, detecting genes enriched in specific physical locations.
- Scanpy's rank_genes_groups applies Wilcoxon rank-sum tests to identify differentially expressed genes between predefined cell groups.
- GiniClust uses Gini index statistics to detect rare cell types through expression dispersion patterns.
- GSPA learns gene embeddings by decomposing expression signals using diffusion wavelets at multiple scales, enabling pattern-based gene clustering across the manifold.

While these methods successfully identify genes marking discrete clusters or spatially enriched regions, they do not distinguish whether localized expression patterns align with the embedding's global geometric structure, e.g., developmental trajectories or continuous cell-state transitions, or instead represent arbitrary spatial subpopulations. This constitutes a substantial methodological gap, particularly because embeddings constructed from transcriptional profiling are often low-dimensional representations of latent cell-state structure and developmental trajectories.

SpectralRH addresses this gap by quantifying expression smoothness and spectral energy concentration in low-frequency Laplacian eigenmodes, explicitly testing whether gene expression patterns follow large-scale manifold geometry. Genes that vary smoothly along trajectories or gradients exhibit high energy in low frequencies, whereas genes marking disconnected clusters or arbitrary regions on the embedding do not, enabling SpectralRH to prioritize trajectory-associated markers over arbitrary localized patterns.

S1.8.2 Algorithm

SpectralRH quantifies how smoothly and coherently each gene's expression varies across the cell-state manifold. Let $X \in \mathbb{R}^{n \times G}$ be a normalized single-cell expression matrix, where n is the number of cells and G is the number of genes. Let $L = I - D^{-1/2} W D^{-1/2}$ be the symmetric normalized Laplacian of the cell-cell connectivity graph (constructed from a k-nearest-neighbor graph in PCA space). Each gene expression profile $x_g \in \mathbb{R}^n$ is treated as a graph signal.

For each gene g, we compute two complementary spectral quantities:

1. **Rayleigh smoothness:**
$$R_g = \frac{x_g^\top L x_g}{x_g^\top x_g}, \tag{10}$$
which measures the graph-smoothness of x_g—i.e., how rapidly expression changes between neighboring cells. Smaller values of R_g correspond to smoother, spatially coherent signals.

2. **Spectral entropy:**
$$H_g = -\sum_{k=1}^{m} p_{gk} \log p_{gk}, \quad p_{gk} = \frac{|\hat{x}_{gk}|^2}{\sum_{\ell=1}^{m} |\hat{x}_{g\ell}|^2}, \tag{11}$$
where $\hat{x}_g = U^\top x_g$ are the coefficients of x_g in the first m eigenvectors U of L (corresponding to the smallest eigenvalues). H_g measures how concentrated the signal's energy is in the low-frequency eigenmodes of the graph; low-entropy signals have compact, structured spatial support.

Because both R_g and H_g depend strongly on the number of expressing cells, we constructed sample-size-matched null distributions. For each observed expression prevalence $k_g = \sum_i \mathbb{I}[x_{ig} > 0]$, we generated random expression patterns with the same sparsity and computed corresponding null values (R_g^0, H_g^0). Observed scores were then converted to empirical p-values or percentiles relative to these nulls, improving comparability across genes with different prevalence.

Finally, for each gene we normalized and averaged the two calibrated metrics to produce a single composite score:

$$\text{SpectralRH}_g = \tfrac{1}{2}\left(\tilde{R}_g + \tilde{H}_g\right), \tag{12}$$

where $\tilde{R}_g$ and $\tilde{H}_g$ denote normalized, size-matched percentiles of Rayleigh smoothness and spectral entropy, respectively. Lower values of the SpectralRH score indicate smoother and more structured expression patterns consistent with spatial localization.

S2 Supplementary Data

CSV files used to populate the split-stability summary tables for the IFN-β stimulation analysis, together with the full ESC time-course localization results table, are included as:

- `data/stim_twothirds_cv_table_across_fold_wide.csv`
- `data/stim_twothirds_cv_table_within_seed_wide.csv`
- `data/stim_twothirds_cv_table_pc_sensitivity_wide.csv`
- `data/esc_locpatterns_dataframe.csv`

S3 Supplementary Figures

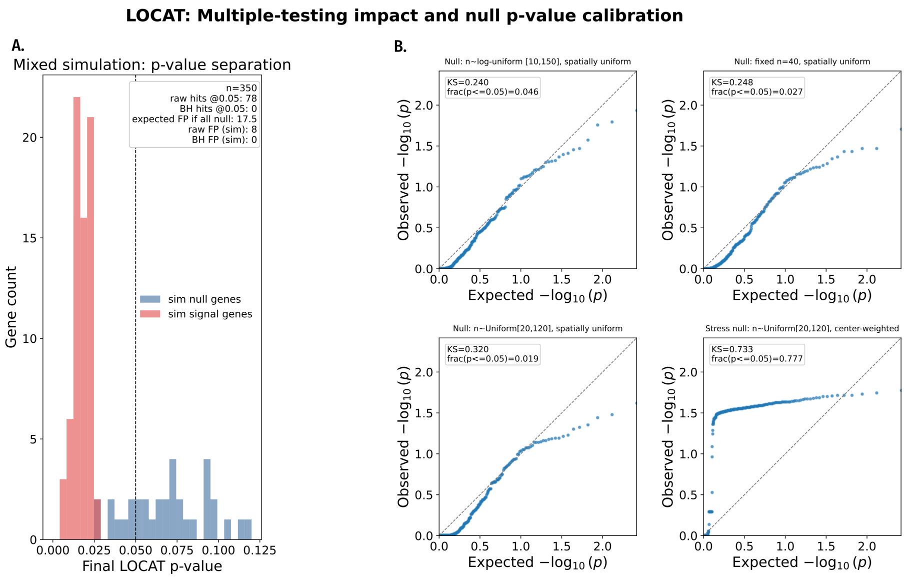

Figure S1: Simulation-based calibration of Locat localization p-values in a synthetic 2D Gaussian embedding (4,500 cells). (**A**) Mixed simulation with 350 genes (70 localized signal, 280 null), with per-gene prevalence sampled between 25 and 89 cells. Signal genes are enriched near the embedding center, whereas null genes are sampled spatially uniformly. The final localization p-values separate signal from null genes, while BH correction is overly conservative and detects zero true positives. (**B**) QQ plots under four null-only settings (260 genes each): fixed $n = 40$, $n \sim \text{Uniform}[20, 120]$, $n \sim$ log-uniform on $[10, 150]$, and a non-uniform center-biased null. Uniform null scenarios are close to nominal calibration (fractions near 0.05 at $p < 0.05$), while the center-biased case shows the largest deviation because null genes are sampled more often near the same central region where signal genes are localized, making this null more conducive to localized-like genes.

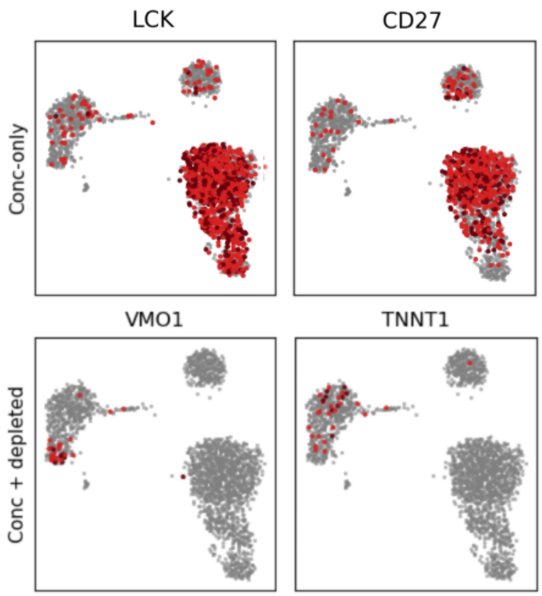

Figure S2: UMAP embeddings for the Scanpy PBMC3k dataset, grouped into concentration-only genes (top) and genes classified as significantly localized by combined concentration and depletion criteria (bottom). Genes in the bottom row show minimal expression outside enriched regions while varying in regional density and compactness.

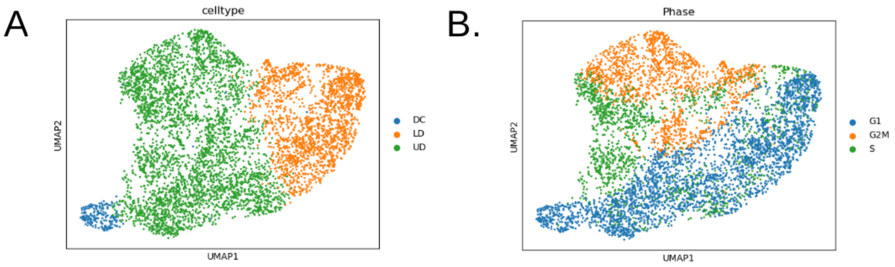

Figure S3: Murine embryonic dermis dataset at E14.5, containing approximately 5,000 cells spanning the transition from upper dermal fibroblasts to Sox2-positive dermal condensates (early hair-follicle precursors). This system forms a continuous transcriptional manifold without discrete cluster boundaries. **(A, B)** t-SNE embedding constructed from 4,000 highly variable genes and labeled by dermal condensate (DC) versus upper dermal (UD) cell type (**A**) and by primary cell-cycle phase (**B**).

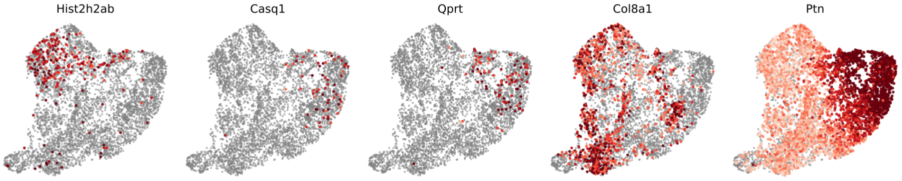

Figure S4: Representative genes with large rank discrepancies between Locat and other methods. Genes ranked highly by Locat but lower by other methods include *Histh2ab* (Locat: 98th percentile; LMD: 94th percentile, scHaystack 74th percentile), *Casq1* (Locat: 99th percentile; GSPA: 88th percentile; LMD: 96th percentile, Hotspot: 64th percentile) and *Qprt* (Locat: 99th percentile; GSPA: 91st percentile; LMD: 96th percentile; Hotspot: 74th percentile). These genes show tightly confined expression with minimal background. In contrast, genes ranked higher by enrichment-focused methods include *Col8a1* (LMD: 93rd percentile; GSPA: 99th percentile, Locat: 76th percentile) and *Ptn* (LMD: 99th percentile, SpectralRH: 98th percentile; Haystack: 99th percentile; Locat: 5th percentile), which show moderate local enrichment with substantial background expression. These diffuse patterns can score highly under concentration-only criteria but are deprioritized by the depletion term in Locat.

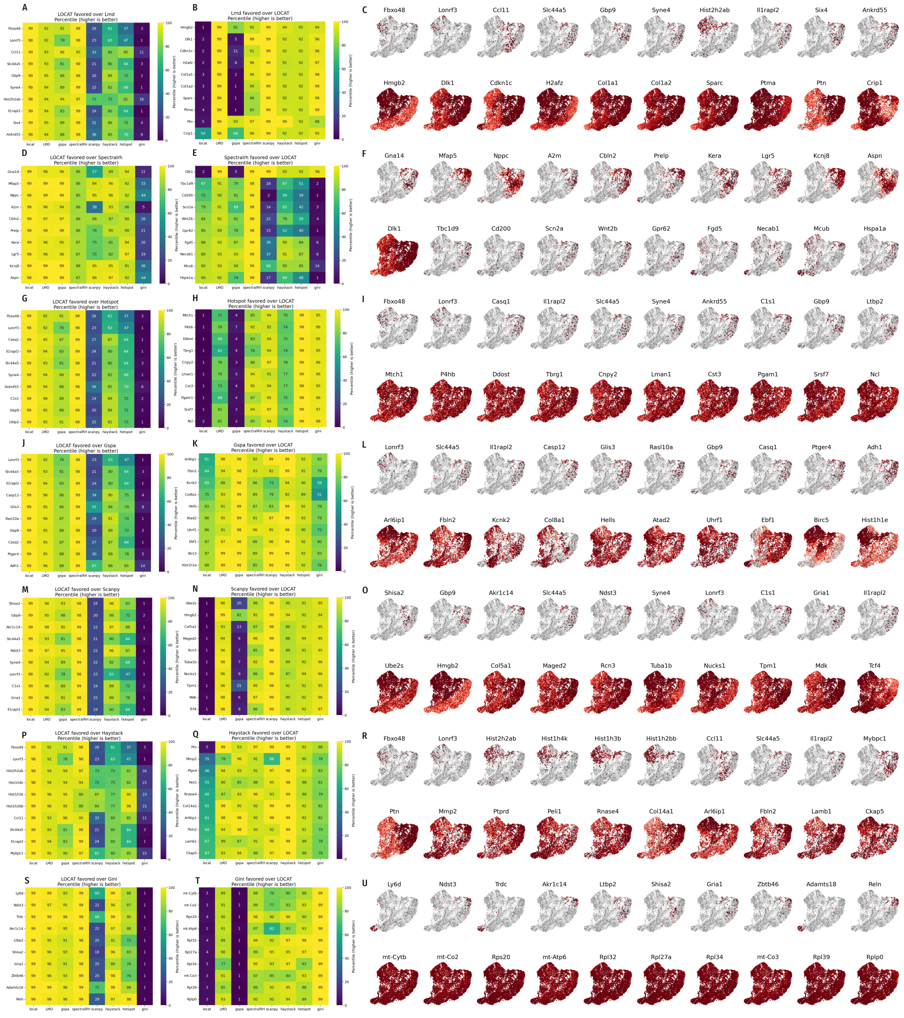

Figure S5: Comparison of Locat with alternative methods using the same gene set and preprocessed murine developmental dermis data. Percentile discrepancies are summarized for each method pair. Panels **A**–**C** show the Locat versus LMD comparison: genes favored by Locat (**A**), genes favored by LMD (**B**), and expression plots for those genes (**C**). The same panel structure is repeated for SpectralRH (**D**–**F**), Hotspot (**G**–**I**), GSPA (**J**–**L**), Scanpy (**M**–**O**), Haystack (**P**–**R**), and Gini (**S**–**U**).

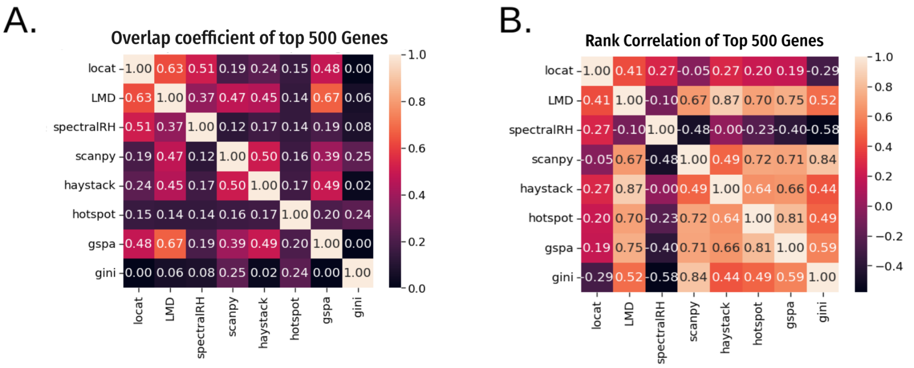

Figure S6: Restricting analysis to the top 500 genes per method still shows that other rankings remain distinct from Locat. **A.** Overlap coefficient for top-500 gene sets across methods; LMD and SpectralRH show the highest overlap with Locat (0.63 and 0.51, respectively). **B.** Correlation matrix computed on top 500 genes ranked by Locat scores; LMD has the highest correlation again in this regime.

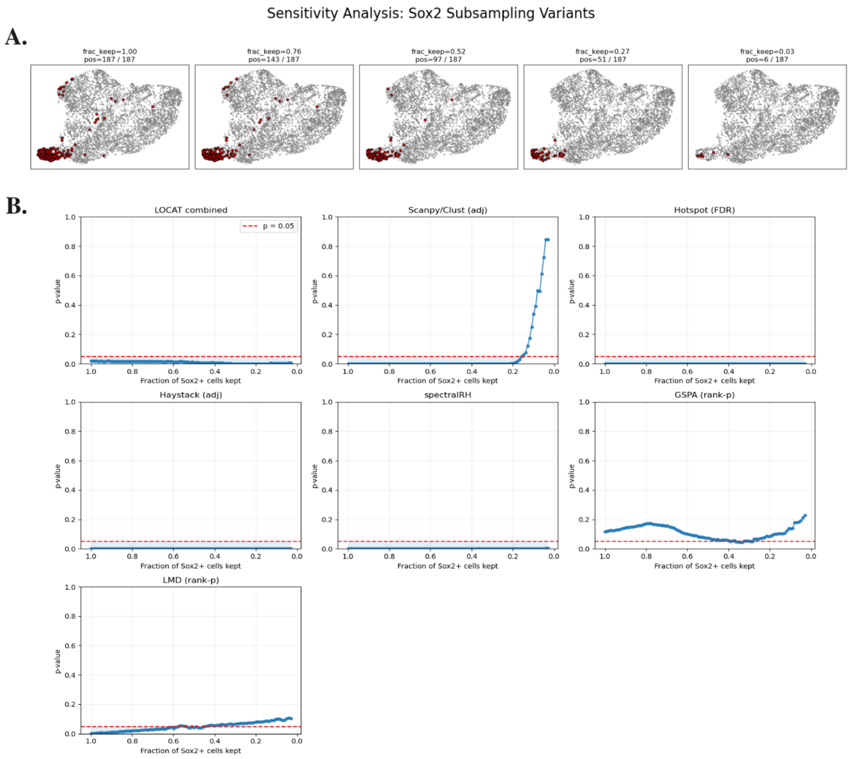

Figure S7: (A) UMAP embeddings of the murine dermis dataset showing simulated *Sox2* expression under progressive subsampling of expressing cells, with the original expression pattern and cell embedding held fixed. (B) Sensitivity of gene prioritization methods as a function of the fraction of *Sox2*-expressing cells retained. Locat maintains detectability across nearly the full range of subsampling levels. SpectralRH, Hotspot, and Haystack show limited sensitivity to reduced prevalence, whereas cluster-based differential expression rapidly loses sensitivity as expressing cells become rare. GSPA and LMD exhibit a gradual degradation of empirical significance values with decreasing prevalence.

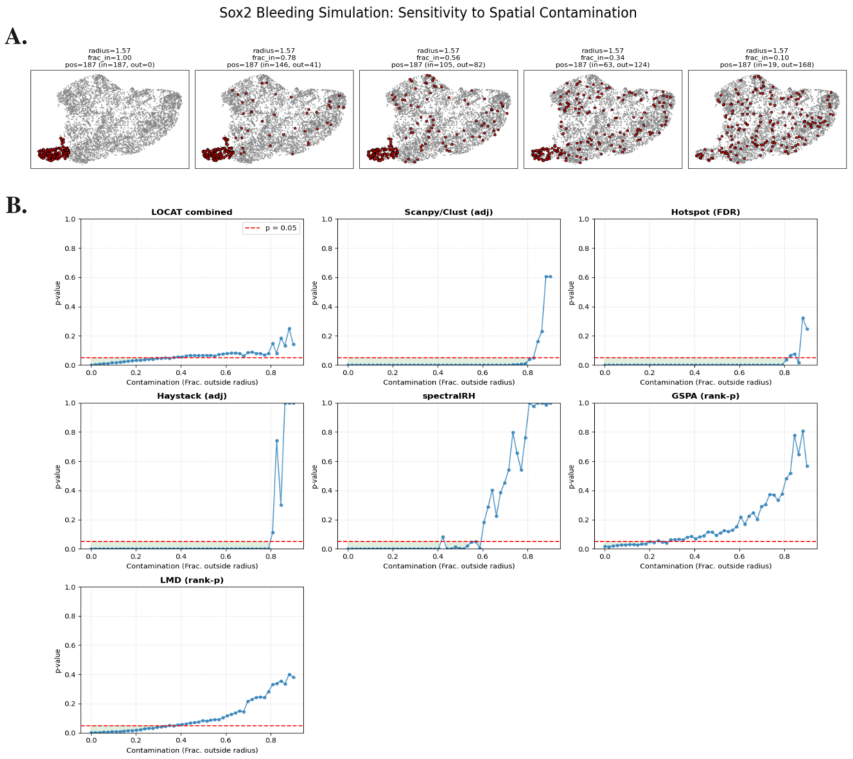

Figure S8: (A) UMAP embeddings showing progressive redistribution of simulated *Sox2* expression into the background population while holding the number of expressing cells fixed. (B) Response of gene prioritization methods to increasing diffusion of *Sox2* expression. Locat shows a graded loss of detectability, with the combined p-value increasing and crossing the detection threshold as expression becomes broadly distributed. SpectralRH, GSPA, and LMD similarly exhibit monotonic score increases with increasing diffusion, while Hotspot, Haystack, and cluster-based differential expression remain largely insensitive until extreme diffusion.

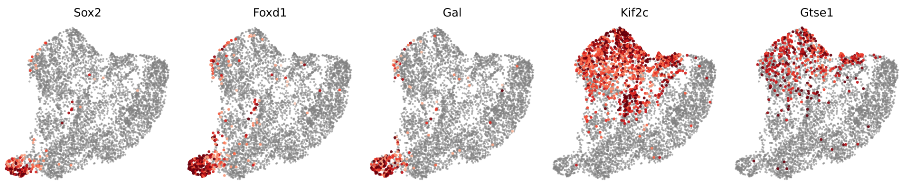

Figure S9: UMAP embeddings colored by expression of representative genes identified as localized by Locat. Dermal condensate-associated genes (*Sox2*, *Foxd1*, *Gal*) localize to terminal regions of the fibroblast-to-dermal condensate trajectory. Cell-cycle-associated genes (*Kif2c*, *Gtse1*) exhibit spatially restricted expression patterns consistent with discrete cell-cycle states.

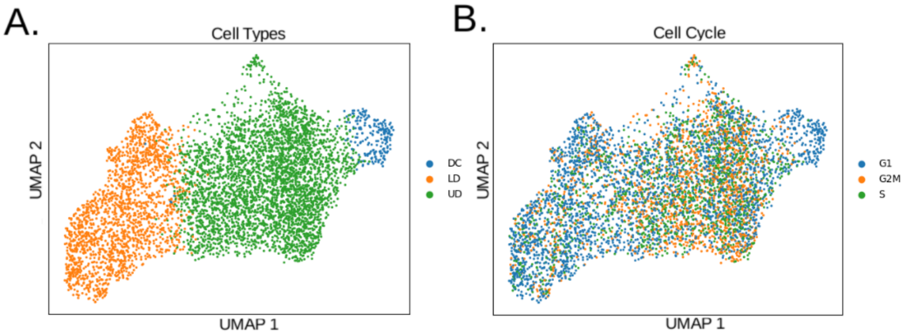

Figure S10: Embedding coordinates computed from a differentiation-focused gene partition in the murine embryonic dermis dataset, as defined in a prior study [2].

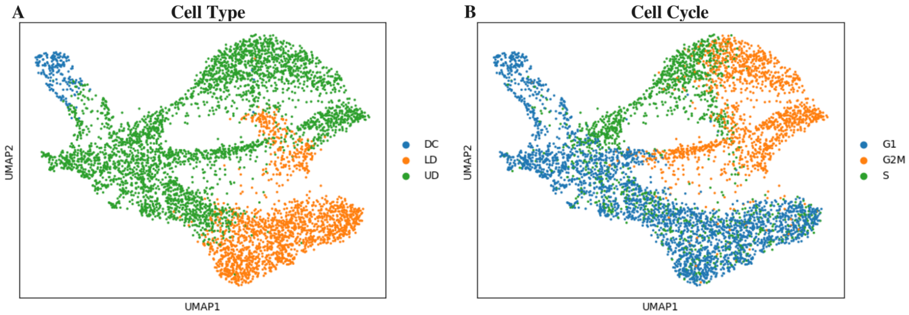

Figure S11: localized-gene embedding (LG-embedding) labeled by (**A**) dermal cell type (UD, LD, DC) and (**B**) cell-cycle phase (G1, G2/M, S). In the murine embryonic dermis dataset, Locat identified 403 genes at $p < 0.05$ with spatially compact expression patterns aligned with proliferative and differentiation-associated variation. This embedding was computed from the significantly localized gene subset.

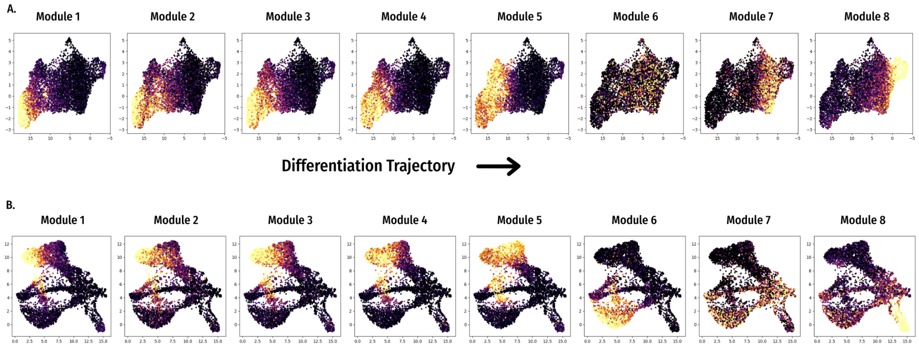

Figure S12: Average module expression for all eight non-singleton localized-gene modules, shown on (**A**) the differentiation-focused UMAP embedding (Fig. S10) and (**B**) the localized-gene embedding (LG-embedding; Fig. S11). Modules were derived from 1,302 significantly localized genes ($\alpha = 0.05$) after training Locat on the murine dermal condensate dataset using UMAP coordinates from a differentiation-focused gene partition [2].

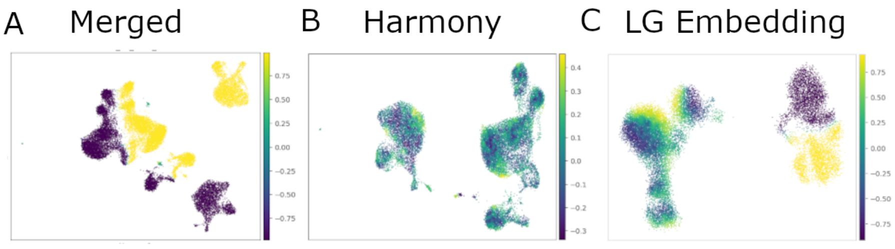

Figure S13: Differential-abundance results projected onto UMAP embeddings generated using (left) a simple merge of preprocessed CTR and STIM samples, (middle) Harmony batch correction, and (right) a simple merge using only localized genes. Batch correction over-smooths the two samples, whereas merging on all genes fully separates them. Using localized genes aligns most shared populations while preserving separation of IFN-responsive populations [3].

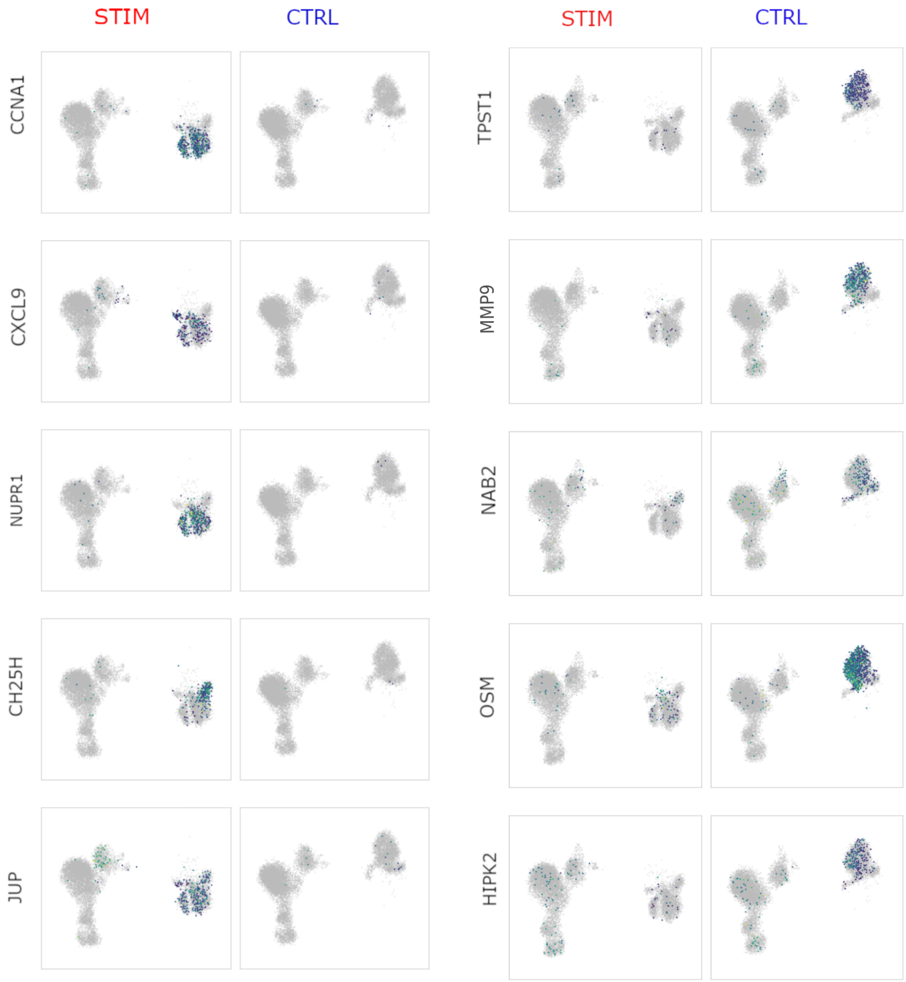

Figure S14: Condition-specific localization examples plotted on a local-gene embedding that aligns CTR and STIM samples from the IFN-β stimulation dataset [3].

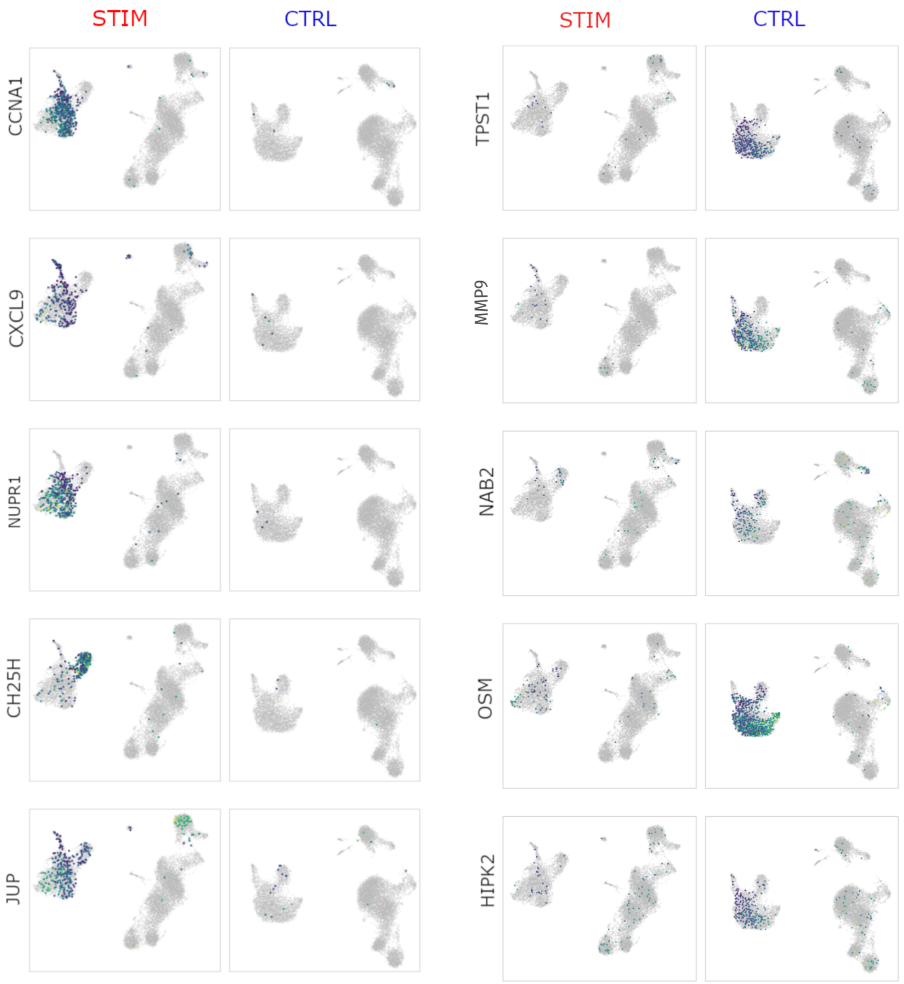

Figure S15: Condition-specific localization examples plotted on per-sample UMAP embeddings from the IFN-β stimulation dataset [3].

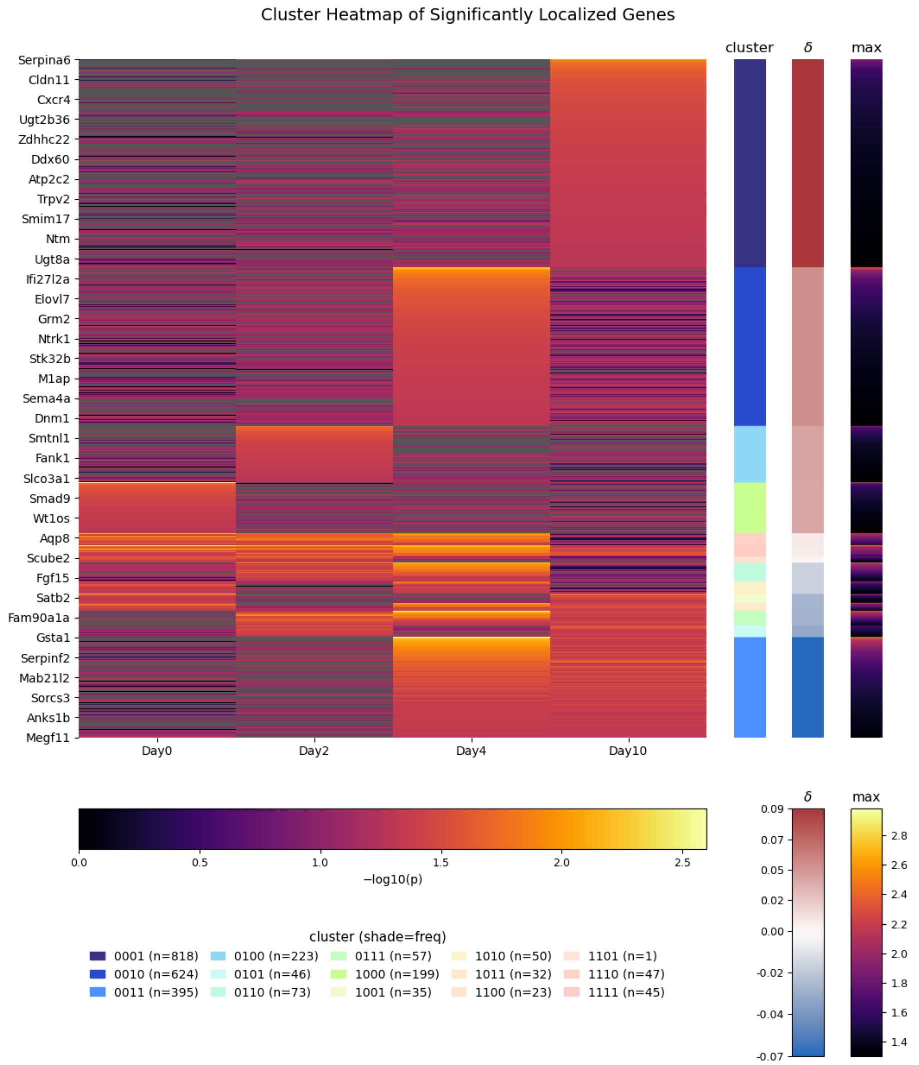

Figure S16: Heatmap of combined localization p-values across time points for all genes (rows) that pass a significance threshold in at least one time point. Colors follow the lower color bar; NA values (unobserved genes) are shown in gray. Genes are grouped by shared temporal localization patterns. The right-side bars report each gene's maximum $-\log_{10}$ localization p-value (overall significance), δ (deviation of observed versus expected pattern frequency), and pattern-cluster identity.

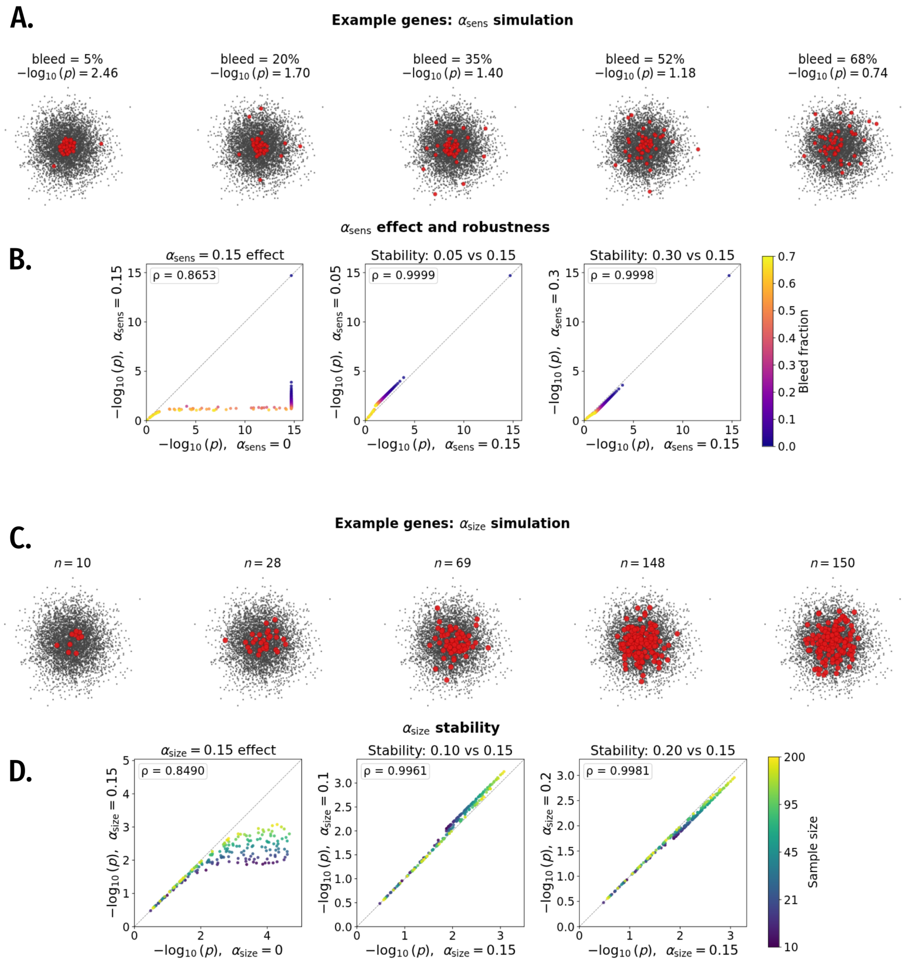

Figure S17: (**A**–**B**) Effect of the sensitivity coefficient (α_{sens}): in a 5,000-cell Gaussian embedding, genes were simulated with fixed total expression ($n = 50$) while varying the fraction of expressing cells located outside a focal enriched region ("bleed," 0–70%). (**A**) shows representative simulated patterns across the bleed range, and (**B**) shows the corresponding $-\log_{10}$ adjusted p-values under varying α_{sens}. Increasing α_{sens} progressively downweights genes with greater bleed, reordering genes according to expression outside enriched regions while preserving overall ranking stability. (**C**–**D**) Effect of the prevalence coefficient (α_{size}): genes were simulated across a range of expressing cell counts (log-uniformly from 200 to 10) within the same embedding framework. (**C**) shows representative simulated patterns across the prevalence range, and (**D**) shows the corresponding $-\log_{10}$ adjusted p-values under varying α_{size}. Increasing α_{size} lowers scores across genes, with stronger reductions for low-prevalence genes, separating genes with similar enrichment strength but differing numbers of expressing cells; rankings remain highly stable across the tested parameter ranges.

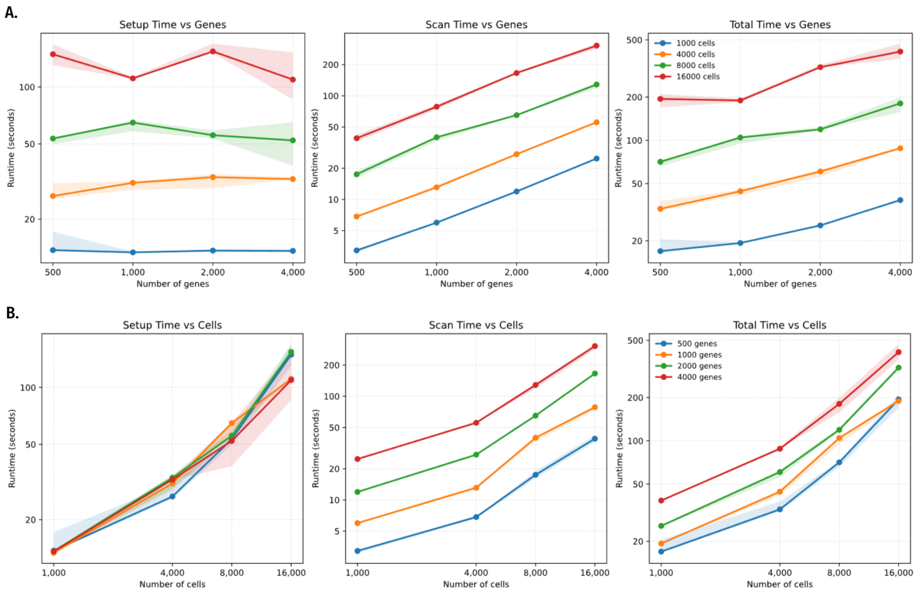

Figure S18: Runtime scaling for Locat using the current `locat_condensed.py` implementation. **A.** Setup, scan, and total runtime versus number of genes (fixed cell count). **B.** Setup, scan, and total runtime versus number of cells (fixed gene count). Benchmarks used three replicates per grid point and report median trends with variability bands.

S4 Supplementary Tables

Table S1: Primary analysis defaults used for Locat (gmm_scan_new).

Parameter	Value	Role
max_freq	0.9	Exclude genes above prevalence threshold from concentration/depletion testing.
rc_weight_mode	binary	Depletion observed fraction uses expressing-cell support (not expression magnitude).
rc_rho_bb	0.02	Beta-binomial overdispersion for depletion tail probability.
rc_lambda_values	[1.0, 2.0] (13 points, geometric)	Contrast-threshold scan grid for depletion.
rc_min_p0_abs	0.10	Minimum background mass in depletion region.
rc_min_expected	3	Minimum expected effective count in depletion region.
rc_min_abs_deficit	0.04	Minimum absolute depletion deficit.
rc_eps_rel	0.01	Relative contrast guard in depletion scan.
rc_n_eff_scale	0.6	Multiplicative scaling applied to Kish effective sample size.
rc_n_trials_cap	$\sqrt{n_{\text{cells}}}$ (rounded to integer)	Upper cap on effective trials for depletion test.
rc_p_floor	10^{-12}	Numerical floor for depletion p-values.
rc_soft_bound	1.0	Optional upper bound on depletion-region background mass ($p0$_abs); active only when set below 1.0.
α_{size}	0.05	Sparsity penalty coefficient in adjusted localization score.
α_{sens}	0.12	Sensitivity penalty coefficient in adjusted localization score.

Table S3: Cross-fold reproducibility of Locat gene localization rankings in the IFN-β stimulation dataset. Two disjoint one-third subsets of stimulated cells were analyzed independently, including PCA recomputation within each subset. For the BiPCA setting, we estimated the effective rank within each fold, used

Table S2: Difference between observed and expected frequencies (δ) for each temporal localization cluster in the time series of embryonic stem cell differentiation following retinoic acid induction. The four digits of each cluster label correspond to the four time points, with the first digit corresponding to Day 0, the second to Day 2, and so on. Each digit is binary, with values of 1 in a given label and digit indicating localization is detected for that cluster at that time point.

cluster label	δ
0001	0.090
0010	0.049
0100	0.039
1000	0.038
1110	0.009
1111	0.007
1100	-0.000
1101	-0.010
0110	-0.017
1010	-0.017
1001	-0.028
1011	-0.029
0111	-0.030
0101	-0.035
0011	-0.065

that number of PCs to construct the neighbor graph, and then computed a UMAP embedding for Locat. The estimated ranks were 47 for fold A and 48 for fold B. The AUROC row reports recovery of significant genes using held-out $-\log_{10}(p+10^{-12})$ scores across the full shared gene panel. Entries report mean metric values with 95% confidence interval half-widths.

**Metric**	**4 PCs**	**8 PCs**	**12 PCs**	**BiPCA→UMAP**
AUROC (all genes; $-\log_{10} p$)	0.923 ± 0.009	0.977 ± 0.004	0.966 ± 0.004	0.979 ± 0.003
Spearman rho (top 200)	0.862 ± 0.104	0.807 ± 0.081	0.728 ± 0.065	0.824 ± 0.064
Spearman rho (top 500)	0.697 ± 0.087	0.762 ± 0.034	0.681 ± 0.009	0.809 ± 0.028
Jaccard overlap (significant genes)	0.694 ± 0.043	0.740 ± 0.030	0.731 ± 0.063	0.734 ± 0.031

Table S4: Within-fold seed stability of Locat gene localization rankings in the IFN-β stimulation dataset. For each fold and embedding setting, Locat was rerun with three random seeds while keeping the embedding fixed; entries summarize all within-fold seed-pair comparisons across the two folds. For the BiPCA setting, we estimated the effective rank within each fold, used that number of PCs to construct the neighbor graph, and then computed a UMAP embedding for Locat. The estimated ranks were 47 for fold A and 48 for fold B. The AUROC row reports recovery of significant genes using held-out $-\log_{10}(p+10^{-12})$ scores across the full shared gene panel. Values report means with 95% confidence interval half-widths.

**Metric**	**4 PCs**	**8 PCs**	**12 PCs**	**BiPCA→UMAP**
AUROC (all genes; $-\log_{10} p$)	0.940 ± 0.013	0.978 ± 0.005	0.969 ± 0.009	0.985 ± 0.007
Spearman rho (top 200)	0.876 ± 0.036	0.832 ± 0.047	0.754 ± 0.065	0.921 ± 0.024
Spearman rho (top 500)	0.748 ± 0.053	0.792 ± 0.012	0.709 ± 0.025	0.874 ± 0.046
Jaccard overlap (significant genes)	0.773 ± 0.036	0.743 ± 0.026	0.769 ± 0.018	0.829 ± 0.040

Table S5: Embedding sensitivity of Locat gene localization rankings in the IFN-β stimulation dataset. Entries summarize within-fold comparisons between embedding settings, pooled across both folds and all seed-matched comparisons. For the BiPCA setting, we estimated the effective rank within each fold, used that number of PCs to construct the neighbor graph, and then computed a UMAP embedding for Locat. The estimated ranks were 47 for fold A and 48

for fold B. The AUROC row reports recovery of significant genes using held-out $-\log_{10}(p+10^{-12})$ scores across the full shared gene panel. Values report means with 95% confidence interval half-widths.

**Metric**	**4 vs 8**	**4 vs 12**	**4 vs BiPCA**	**12 vs BiPCA**
AUROC (all genes; $-\log_{10} p$)	0.934 ± 0.014	0.921 ± 0.030	0.933 ± 0.016	0.951 ± 0.024
Spearman rho (top 200)	0.778 ± 0.035	0.684 ± 0.057	0.758 ± 0.047	0.748 ± 0.040
Spearman rho (top 500)	0.673 ± 0.026	0.592 ± 0.031	0.685 ± 0.073	0.682 ± 0.040
Jaccard overlap (significant genes)	0.526 ± 0.029	0.435 ± 0.046	0.561 ± 0.035	0.624 ± 0.055

S5 Supplementary Notes

References

[1] I. D. Gebru, X. Alameda-Pineda, F. Forbes, and R. Horaud, "Em algorithms for weighted-data clustering with application to audio-visual scene analysis," *IEEE Transactions on Pattern Analysis and Machine Intelligence*, vol. 38, no. 12, pp. 2402–2415, 2016.

[2] E. Peterfreund, O. Lindenbaum, Y. Kluger, and B. Landa, "Partition first, embed later: Laplacian-based feature partitioning for refined embedding and visualization of high-dimensional data," *OpenReview.net*, 2022.

[3] H. M. Kang, M. Subramaniam, S. Targ, M. Nguyen, L. Maliskova, E. McCarthy, E. Wan, S. Wong, L. Byrnes, C. M. Lanata, R. E. Gate, S. Mostafavi, A. Marson, N. Zaitlen, L. A. Criswell, and C. J. Ye, "Multiplexed droplet single-cell rna-sequencing using natural genetic variation," *Nature Biotechnology*, vol. 36, pp. 89–94, 1 2018.
